# Surgically treated ankle fractures in Sweden: a 15-year population-based study of 96 015 surgeries

**DOI:** 10.1186/s12891-025-08414-4

**Published:** 2025-02-18

**Authors:** Gustav Nilsson, Michael Axenhus

**Affiliations:** 1 Department of Orthopaedic Surgery, Danderyd Hospital, Stockholm, Sweden; 2 Department of Clinical Sciences, Danderyd Hospital, Stockholm, Sweden; 3 Orthopaedic clinic, Danderyd University Hospital, Entrévägen 2 182 68, Danderyd, Sweden

**Keywords:** Ankle fracture, Population-based study, Surgery, Sweden, Trends

## Abstract

**Background:**

Ankle fractures are the third most common fractures, often requiring surgical intervention to restore function and mobility. Understanding trends in ankle fracture surgeries is essential for optimizing treatment strategies and improving patient outcomes. The aim of this study is to provide a comprehensive analysis of ankle fracture surgeries in Sweden in order to highlight changes in demography and trends in surgical procedures.

**Methods:**

Utilizing data from the National Patient Register, we conducted an observational population-based study of ankle fracture surgeries performed in Sweden between 2008 and 2022. Patients aged 15 years and above who underwent ankle fracture surgery were included in the analysis. Surgical procedures were identified using NOMESCO codes specific to ankle fractures. Demographic trends, surgical procedures and incidence rates were analyzed using descriptive statistics, incidence calculations and regression analyses.

**Results:**

Women accounted for 55% of surgeries (*p* = 0.022), with a significant proportion (47%) occurring in individuals aged 65 and above. Although the overall incidence of ankle surgeries decreased by 6% (*p* = 0.008), notable age-specific trends emerged, including a decrease in surgeries among younger adults and an increase among the elderly. Plate and screw fixation remained the most commonly employed surgical technique, with a 21% increase in usage (*p* < 0.001), while the use of external fixation and intramedullary nailing increased significantly by 123% and 69%, respectively (both *p* < 0.001). Conversely, the use of cerclage and/or pin fixation decreased by 74% (*p* < 0.001) over the study period.

**Conclusions:**

Our study of open source data shows current trends in surgically treated ankle fractures in Sweden, highlighting a decreased incidence overall, notable shifts between age groups and several trends in surgical procedures. Despite limitations inherent to retrospective observational studies, such as the inability to establish causal relationships, our findings contribute to the understanding of ankle fracture management trends, highlighting areas for further investigation and improvement in orthopedic care.

## Background

Ankle fractures are common injuries affecting both sexes and all age groups [1–3]. In the younger population ankle fractures more commonly are a result of a high energy trauma with male dominance, in comparison to the elderly where the fracture often is a result of a low energy trauma and affecting women [1, 4, 5]. While some ankle fractures can be successfully treated by conservative measures, others require surgical intervention in order to restore joint stability and reduce the risk of post-traumatic arthritis [6–8]. Some studies published in the last few years have indicated a trend of increased incidence of ankle fractures as well as a shift towards surgical treatment [9–11].

All orthopedic departments in Sweden register surgically treated ankle fractures in a national wide diagnosis register, the National Patient Register. The use of national wide diagnosis registers has been proved to be validated for the tracking and study of fractures [12]. The understanding of trends in surgically treated ankle fractures is essential for optimizing treatment strategies and improving patient outcomes. The primary aim of this study is to fill this gap by providing a detailed analysis of trends in surgically treated ankle fractures in Sweden over a 15-year period, from 2008 to 2022. By examining demographic shifts, incidence rates, and the utilization of different surgical techniques, this study seeks to highlight changes in clinical practice and identify patient groups at greater risk of requiring surgical intervention. Furthermore, by understanding these trends, we aim to inform future treatment guidelines and enhance the overall management of ankle fractures.

## Methods

### Study design

This is an observational population-based register study based on data derived from the National Patient Register (NPR) from 2008 to 2022 [13]. The study is conducted in accordance with the RECORD guidelines [14].

### Setting

The Swedish National Health Service ensures healthcare access for all Swedish citizens, offering subsidized emergency treatment, general hospital care, and outpatient visits. Residents of Sweden are assigned a permanent Swedish personal identification number, which is unique and remains associated with them until death or emigration. The personal identification number is used in all interactions with public or private healthcare and is utilized in all national healthcare registers.

### Data source

The NPR has accommodated inpatient care since 1964 (nationwide since 1987) and specialized outpatient care has been included since 2001. The registry receives updates with late-arriving or corrected information even after publication. The NPR contains information about surgical procedures and detailed information about surgical procedures such as geographical distribution, age groups and sex. All hospitals, whether public or private, are required to report data. This includes diagnosis codes based on the International Statistical Classification of Diseases version 10 (ICD-10) since 1994, as well as surgical procedure codes following the classification system of NOMESCO. All *orthopaedic* departments (*n* = 54) in Sweden, who perform fracture surgery, are engaged in the NPR, which represent a 100% coverage.

### Patients

All individuals aged ≥ 15 years at the time of surgery who experienced an ankle fracture (ICD S82) and were recorded in the NPR from 1st of January 2008 to 31st of December 2022, were considered for inclusion. Only persons with a Swedish identification number were extracted. All ankle surgeries due to fracture were extracted from the NPR using the NOMESCO codings for ankle fracture surgery (Code: NHJ). We included all procedural codes for surgical treatments. (Table 1). 

**Table 1:** Included NOMESCO codes regarding surgically treated ankle fractures

Metod	Code
External fixation	NHJ20,21,22,23,29
Bioimplant	NHJ30,31,32,33,39
Cerclage or pinning	NHJ40,41,42,43,49
Intramedullary nailing	NHJ 50,51,52,53 59
Plate fixation with screws	NHJ 60,61,62,63,69
Other or combined method	NHJ 80,81,82,83,89,90,91,92,93,99

### Variables

The incidence data were age-stratified, using 5-year age categories. The treatment distribution was divided into two age groups: 15–64 years for non-elderly patients and 65 + years for elderly patients. Sex was categorized as male or female. Operative treatment was divided into plate and/or screw fixation, cerclage and/or pin fixation, external fixation, fixation with bioimplant, or other combined methods of fracture surgery.

### Statistics

Descriptive statistics were utilized and presented in total numbers and percentages. The incidence was calculated per 100,000 inhabitants where the year specific population data was obtained from Statistics Sweden. Regression analyses were conducted for all treatment methods, considering exponential, linear and polynomial regression models to find the best fit for each incidence trend. Analyses were performed using SPSS (v27.0) (IBM, New York, USA)), Microsoft Excel (v16.77.1) and R v4.0.6 (R Foundation for Statistical Computing, Vienna, Austria). When calculating the number of patients, each unique personal identification number has been counted only once per year, operation, and region.

### Ethics

The study was performed using open-source data and was therefore not subject to ethical review.

## Results

### Descriptive data and patients

In total 96,015 patients who had undergone surgical procedures due to ankle fractures between 2008 and 2022 were derived from the NPR and included for analysis. The majority were women (55%) (*p* = 0.022) and 47% of all ankle surgeries occurred in the age group 65+. Over the course of the 15-year study period there was an increase of 5,4% surgical procedures in absolute numbers from 6,205 cases in 2008 to 6,542 cases in 2022 (*p* = 0.012) (Table 2).

**Table 2:** Number of patients undergoing ankle surgeries in Sweden (2008–2022)

	2008	2009	2010	2011	2012	2013	2014	2015	2016	2017	2018	2019	2020	2021	2022	Total
Total	6,205	6,579	7,018	7,115	6,652	6,841	6,069	6,069	6,081	6,168	6,401	6,206	5,639	6,430	6,542	96,015
Sex																
Men	2,879	3,005	3,184	3,252	3,101	3,119	2,697	2,741	2,746	2,736	2,847	2,808	2,437	2,739	2,805	43,096
Women	3,326	3,574	3,834	3,863	3,551	3,722	3,372	3,328	3,335	3,432	3,554	3,398	3,202	3,691	3,737	52,919
Age																
15–19	498	468	431	401	380	338	363	307	293	296	327	313	322	313	328	5,378
20–24	392	396	481	464	466	441	429	385	385	395	390	341	328	330	353	5,976
25–29	326	318	394	367	337	362	367	375	387	387	430	433	356	330	338	5,507
30–34	317	316	329	377	310	350	287	313	323	320	325	352	343	400	398	5,060
35–39	354	387	411	436	373	352	310	345	337	349	333	342	323	370	386	5,408
40–44	480	518	480	523	466	472	399	429	429	402	388	377	323	424	387	6,497
45–49	477	538	564	667	569	568	516	499	458	467	449	476	418	465	471	7,602
50–54	579	603	677	686	598	641	568	566	566	587	619	575	489	573	553	8,880
55–59	614	622	710	727	665	682	538	526	570	578	596	573	551	663	679	9,294
60–64	692	769	795	717	658	742	604	600	550	582	623	573	549	607	608	9,669
65–69	516	582	657	671	688	703	598	584	603	564	573	530	539	578	553	8,939
70–74	343	391	409	407	465	477	445	476	502	531	548	508	460	524	550	7,036
75–79	236	279	270	271	280	295	281	271	302	330	376	381	312	423	479	4,786
80–84	194	197	204	201	194	204	181	172	193	171	219	185	154	221	250	2,940
85+	187	195	206	200	203	214	183	221	183	209	205	247	172	209	209	3,043

The Swedish population increased by approximately 14% during the study period, predominantly due to an increase of the elderly population. These numbers translate to a mean ankle surgery incidence of 79/100,000/year, corresponding to a decrease of 6% (*p* = 0.008) from 81/100,000 in 2008 to 76/100,000 in 2022. This decrease was especially notable among those aged 15–29, however all age groups aged 15–69 did in fact display a decreased incidence, excluding those aged 30–35 where the incidence was stationary.

**Fig. 1:**
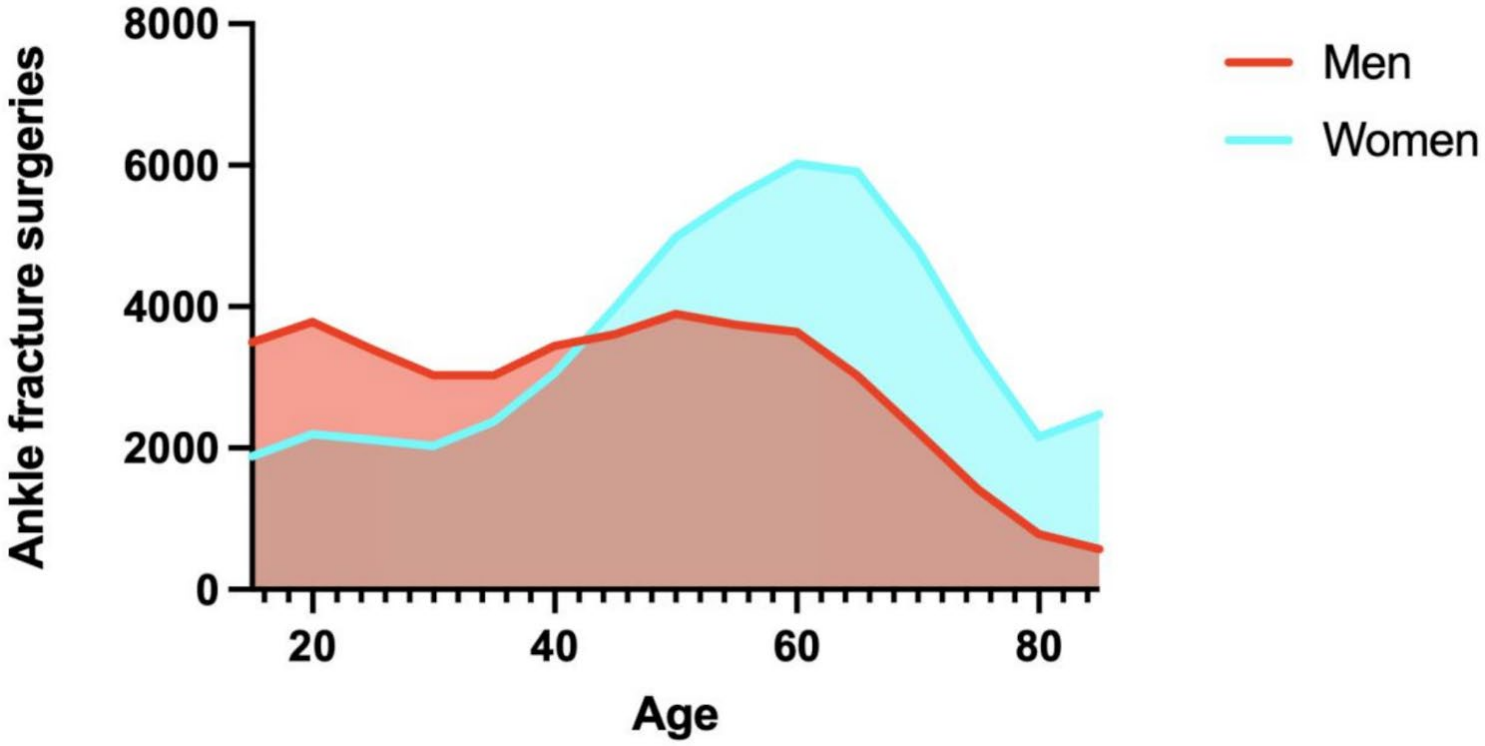
Age and sex distribution of ankle fracture surgeries. Surgeries are per number and sex

Women had a slight increase of 1% (*p* = 0.33) in incidence of ankle fracture surgeries while surgeries among men decreased by 15% (*p* = 0.012) in 2022 compared to 2008. Men were overrepresented in ankle surgeries up until the 5th decade of life. Women and a biphasic distribution with an increase from the 3rd decade of life up until the 7th decade. Ankle surgeries in women and men both decrease from the 7th decade of life (Table 3; Fig. 1).

**Table 3:** Incidence of ankle fracture surgeries in Sweden (2008–2022)

	2008	2009	2010	2011	2012	2013	2014	2015	2016	2017	2018	2019	2020	2021	2022
Total	81	85	90	90	84	86	76	75	74	75	77	73	66	75	76
Sex															
Men	76	78	82	83	79	79	68	68	67	66	68	66	57	64	65
Women	86	91	97	97	89	93	83	82	81	83	85	81	75	86	87
Age															
15–19	78	73	69	66	66	61	68	59	56	55	59	56	56	54	55
20–24	69	67	78	72	71	66	64	58	59	63	64	58	56	57	61
25–29	58	56	68	62	56	58	57	56	56	54	58	59	49	47	50
30–34	54	54	57	65	53	59	48	51	51	49	48	50	47	53	52
35–39	57	61	65	69	60	57	51	57	55	56	53	53	49	55	56
40–44	71	78	73	81	72	74	62	66	66	62	61	60	51	67	60
45–49	80	88	89	102	85	84	76	75	69	71	68	72	63	70	72
50–54	99	103	116	117	102	108	93	89	86	87	90	84	73	86	84
55–59	104	107	124	127	116	118	93	91	98	99	101	94	87	102	101
60–64	111	123	128	118	111	128	107	107	98	103	110	101	97	107	106
65–69	110	117	124	119	117	117	100	98	104	100	103	97	100	107	102
70–74	95	105	107	103	113	110	96	96	96	97	98	90	83	96	104
75–79	77	92	89	89	91	93	86	80	86	90	97	92	71	90	98
80–84	78	80	83	82	79	84	75	71	79	69	85	69	56	77	83
85+	77	79	83	79	80	84	72	86	71	80	79	94	65	79	77

### Treatment

**Fig. 2:**
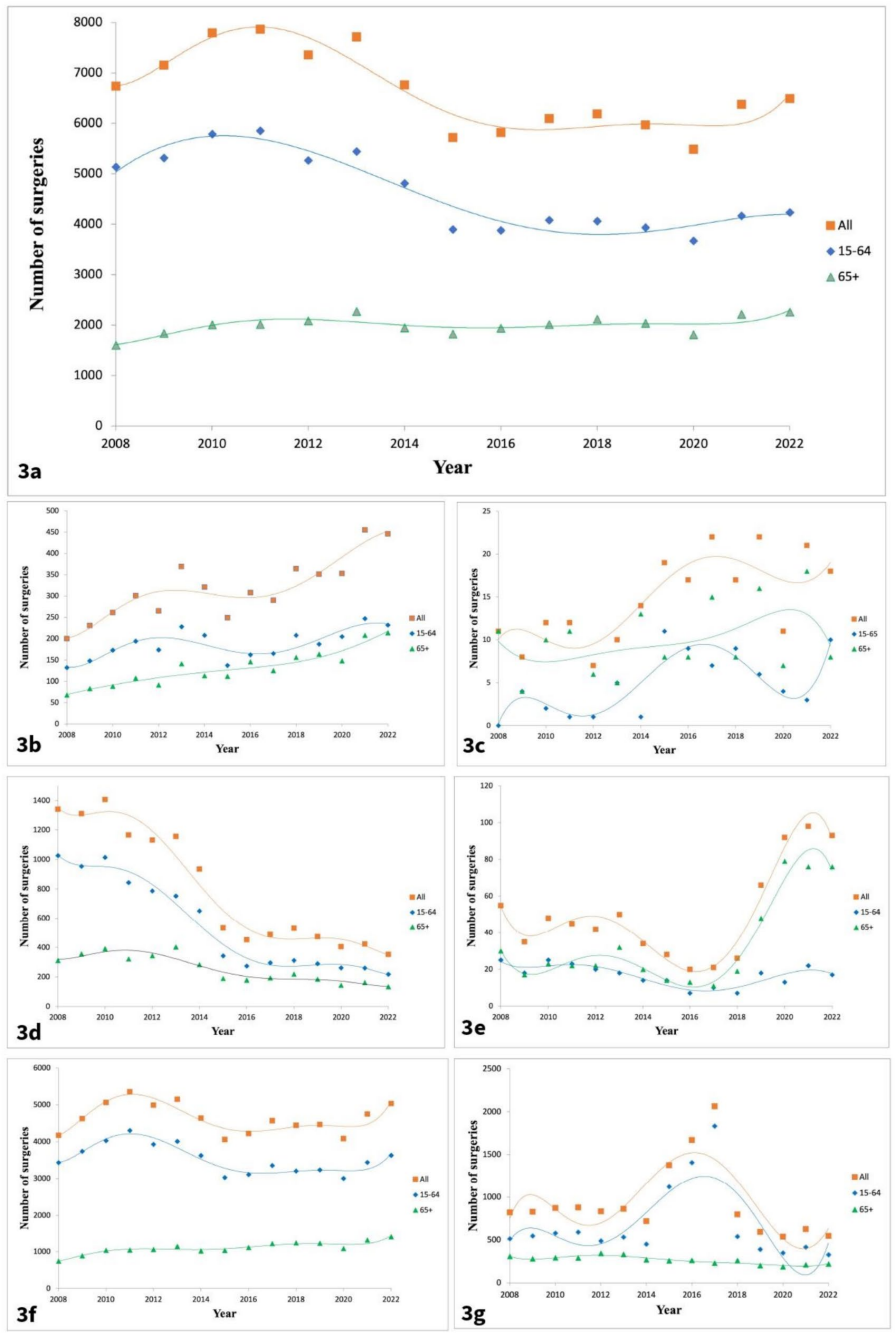
Number of ankle fracture surgical procedures (2008–2022) (**3a**) All treatments (2008–2022), (**3b**) Exfix, (**3c**) Bioimplant, (**3d**) Cerclage and/or pin, (**3e**) Intramedullary nail, (**3f**) plate and/or screws, (**3g**) Other/combined method

During the 15-year study period a total of 99,487 surgical procedures for ankle fractures were performed, ranging from 6,737 in 2008 to 6,492 in 2022, corresponding to a 3,7% decrease (*p* = 0.011). In the 15–64 age group, ankle fracture surgery decreased from 8,168 in 2008 to 6,281 in 2022 (*p* = 0.004), while in the 65 + age group, there was an increasing trend in ankle fracture surgeries from 2,509 in 2008 to 3,276 in 2022 (*p* = 0.003). (Table 4; Fig. 2a).

**Table 4:** Surgical treatment of ankle fractures. Per procedure and age groups (2008–2022)

	2008	2009	2010	2011	2012	2013	2014	2015	2016	2017	2018	2019	2020	2021	2022	Total
All treatments																
All	6,737	7,151	7,792	7,863	7,354	7,710	6,758	5,714	5,815	6,092	6,180	5,970	5,483	6,376	6,492	99,487
15–64	5,134	5,315	5,783	5,852	5,265	5,439	4,812	3,893	3,877	4,077	4,062	3,930	3,672	4,163	4,234	69,508
65+	1,603	1,836	2,009	2,011	2,089	2,271	1,946	1,821	1,938	2,015	2,118	2,040	1,811	2,213	2,258	29,979
Exfix																
All	200	231	261	301	265	369	321	249	308	290	364	351	353	455	446	4,764
15–64	132	148	173	194	174	228	208	137	162	165	208	187	205	247	232	2,800
65+	68	83	88	107	91	141	113	112	146	125	156	164	148	208	214	1,964
Bioimplant																
All	11	8	12	12	7	10	14	19	17	22	17	22	11	21	18	221
15–64	0	4	2	1	1	5	1	11	9	7	9	6	4	3	10	73
65+	11	4	10	11	6	5	13	8	8	15	8	16	7	18	8	148
Cerclage and/or pin																
All	1,341	1,312	1,407	1,167	1,133	1,157	936	534	453	490	532	475	406	424	353	12,120
15–64	1,028	955	1,015	845	788	753	652	344	274	296	312	290	262	261	218	8,293
65+	313	357	392	322	345	404	284	190	179	194	220	185	144	163	135	3,827
Intramedullary nailing																
All	55	35	48	45	42	50	34	28	20	21	26	66	92	98	93	753
15–64	25	18	25	23	20	18	14	14	7	10	7	18	13	22	17	251
65+	30	17	23	22	22	32	20	14	13	11	19	48	79	76	76	502
Plate and/or screws																
All	4,175	4,628	5,067	5,351	4,989	5,154	4,642	4,062	4,221	4,572	4,444	4,463	4,085	4,752	5,036	69,641
15–64	3,433	3,741	4,030	4,307	3,928	4,011	3,624	3,029	3,109	3,351	3,204	3,235	3,003	3,437	3,630	53,072
65+	742	887	1,037	1,044	1,061	1,143	1,018	1,033	1,112	1,221	1,240	1,228	1,082	1,315	1,406	16,569
Other/Combined method																
All	821	827	871	878	832	862	719	1,377	1,670	2,063	797	593	536	626	546	14,018
15–64	513	546	578	589	488	531	451	1,120	1,408	1834	538	391	349	415	326	10,077
65+	308	281	293	289	344	331	268	257	262	229	259	202	187	211	220	3,941

The most popular treatment for ankle fractures was plate and/or screws, with 69,641 surgeries performed in total, a percentual increase of 21% when comparing 2008 to 2022 (*p* < 0.001).

**Fig. 3:**
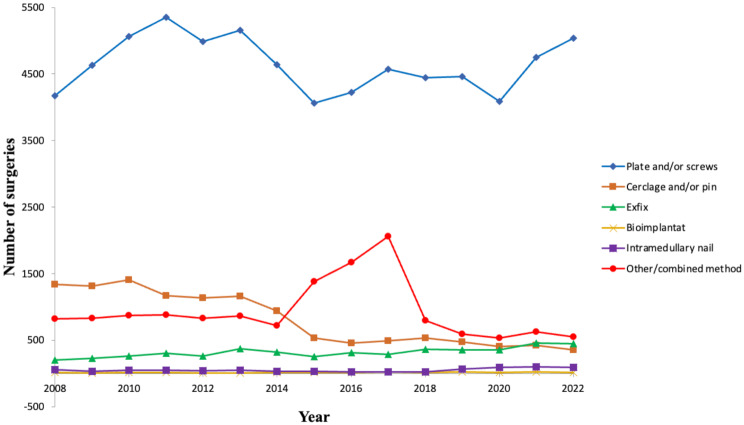
Number of ankle fracture surgical procedures. Per procedure and age groups (2008–2022)

The number of patients aged 65 + receiving this treatment almost doubled, from 742 in 2008 to 1,406 in 2022. The age group 15–64 showed a pendulous yearly variance of the number of surgeries without a clear trend. (Table 4; Figs. 3 and 2f).

Exfix procedures more than doubled with an increase from 200 in 2008 to 446 in 2022 (*p* < 0.001). Intramedullary nailing also displayed a significant increase of 69% (*p* < 0.001) due to increased utilization in the 65 + age group, who accounted for 67% of all intramedullary nails surgeries in total (Table 4; Fig. 2c, e).

Bioimplant surgeries remained relatively stable, ranging from 11 in 2008 to 21 in 2022, and were mainly utilized in the 65 + age group. Cerclage and/or pin surgeries decreased from 1,341 in 2008 to 353 in 2022 (*p* < 0.001). The main decrease was seen within the age group 15–64. Lastly, surgeries involving other/combined methods ranged from 821 in 2008 to 282 in 2022 (*p* < 0.001). (Table 4; Fig. 2c-d, g)

## Discussion

The findings of our study reveal several notable trends in ankle fracture surgeries in Sweden over the 15-year period examined. The overall decrease in the incidence of ankle surgeries by 6% contrasts with a slight increase in the total number of surgeries due to population growth, particularly in the elderly. Only among those aged 70 years and above the incidence increased. These alterations could be attributed to various factors, including changes in guidelines of conservative vs. surgical treatment, improved osteosynthesis materials and surgical techniques as well as a more vital elderly population considered suitable for surgery [15–17]. Another possible explanation is the aging population demographic observed in Sweden, where the number of citizens aged 60 and above increased by 30% during the study period. The aging population may have contributed to the increased number of ankle surgeries, as older individuals are more prone to fractures due to increased prevalence of osteoporosis and increased risk of falls [18, 19]. However, other studies have shown that similar demographic changes in some cases can’t explain the increase in ankle fractures and further research is warranted [11]. There is no published data on the total number of ankle fractures in Sweden between 2008 and 2022, surgically treated and non-surgically treated. Alterations regarding the total number of ankle fractures might also be a factor influencing the results. Other studies evaluating the incidence of surgically treated ankle fractures are sparse. One study over a 10-year period in England between 2007 and 2017 has indicated a relatively stable incidence of surgically treated ankle fractures for both younger and older patients [20].

Demographically, our study highlights a higher prevalence of ankle surgeries among women, who accounted for 55% of all cases. Men were overrepresented in ankle surgeries up until the 5th decade of life and women had a peak incidence at approximately 50 to 70 years old. Other studies have shown similar age and sex distribution [1]. This is likely influenced by the higher life expectancy of women and their greater likelihood of osteoporosis, which exacerbates fracture risk [18, 19]. The incidence of ankle fracture surgery for female sex increased by 1% between 2008 and 2022, conversely for male sex the incidence decreased by 15%. The demographic changes explained in the previous paragraph could be a contributing factor to this. With an increasing elderly population, where women suffer more fractures than men, a gender gap is expected.

In terms of surgical treatments, plate and/or screw fixation emerged as the most popular treatment modality for ankle fractures, with 69,641 surgeries performed over the study period. Cerclage and/or pin surgeries decreased from 1,341 in 2008 to 353 in 2022, a decrease of 74%, mainly due to a decrease in those aged 15–64. Both external fixation and intramedullary nails increased significantly, 123% and 69% respectively, but from low absolute numbers. Regarding intramedullary nails, the increase is mainly seen among the elderly and after 2017. These findings may reflect new guidelines regarding treatment of choice, improved osteosynthesis materials and may also be influenced by factors such as alterations in fracture severity and patient comorbidities. The observed fluctuations in surgical treatments underscore the need for ongoing evaluation of treatment efficacy and outcomes. An English study with more than 200 000 ankle fractures over a 10-years period between 2007 and 2017 reports a stable incidence of internal fixation, but an increased use of intramedullary implants of 15–20% [20]. The significant decrease in the use of cerclage and/or pinning likely reflects a consensus on their lower efficacy or greater complication rates, leading to their phased-out usage in favor of more robust fixation methods.

The age group 15–19 is heterogeneous, including patients both before and after physeal closure, as this occurs in the distal tibia and fibula around 12–15 years for females and 15–20 years for males [21]. However, in Sweden, patients aged ≥ 15 years are predominantly treated by non-pediatric orthopedic departments and most pediatric ankle fractures occur before the age of 15 during growth plate fusion [22]. Thus, the cut-off age was set to 15 years in inorder to study trends representative to the treated population at the orthopedic departments.

To our knowledge this is the first study investigating the trends in surgically treated ankle fractures in Sweden, providing valuable large-scale data with a 100% coverage. The trends observed in this study suggest several implications for clinical practice. The increased incidence in surgeries among the elderly highlights the importance of proactive measures in this population, such as fall prevention programs and bone health optimization, which could reduce the need for surgical intervention. For younger populations, where a decrease in surgeries was observed, further research into the effectiveness of conservative treatments and their long-term outcomes is warranted. Additionally, the evolving landscape of surgical techniques calls for ongoing assessment of their comparative effectiveness. Randomized controlled trials (RCTs) or large-scale cohort studies could help clarify the long-term outcomes associated with different surgical methods, particularly newer approaches like intramedullary nailing, compared to traditional plate and screw fixation.

The retrospective nature of our study design limits our ability to establish causal relationships. Furthermore, the study is limited to surgically treated ankle fractures hindering comparisons to simultaneous trends in ankle fractures non-surgically treated. Another limitation is that coded data has been shown to contain errors, for example one study found that the primary diagnosis was incorrect in 13% of evaluated cases [23]. Also, the data derived from the NPR does not allow for some analyses, for example how many patients were definitely managed in external fixation or breakdown of intramedullary nails into hindhood vs. fibular nails. While we observed several trends in both the epidemiology and treatment of surgically treated ankle fractures, further studies are needed to confirm these findings and explore potential confounding factors such as fracture severity.

In conclusion our study offers a comprehensive analysis of trends in ankle fracture surgeries in Sweden over a 15-year period. We observed a decreased incidence of ankle surgeries in total, but major shifts between age groups where the incidence increased among the elderly and decreased for the younger population. Regarding surgical procedures several trends emerged. Cerclage and/or pin decreased significantly, while both exfix and intramedullary nail increased and the plate and/or screw fixation maintained its position as the most used method. Our study highlights areas for further investigation and improvement in ankle fracture management trends.

## Data Availability

The data sets can be obtained from the NPR directly (https://www.socialstyrelsen.se/en/statistics-and-data/statistics/statistical-databases/).
